# *ABCA1* C69T Gene Polymorphism Association with Dysglycemia in Saudi Prediabetic Adults

**DOI:** 10.3390/genes13122277

**Published:** 2022-12-02

**Authors:** Ghada M. A. Ajabnoor, Suhad M. Bahijri, Wafa Alrashidi, Sumia Mohammad Enani, Aliaa A. Alamoudi, Lubna Al Sheikh, Basmah Eldakhakhny

**Affiliations:** 1 Department of Clinical Biochemistry, Faculty of Medicine, King Abdulaziz University, Jeddah 21551, Saudi Arabia; 2 Saudi Diabetes Research Group, King Fahd Medical Research Centre, King Abdulaziz University, Jeddah 3270, Saudi Arabia; 3 Food, Nutrition and Lifestyle Unit, King Fahd Medical Research Center, King Abdulaziz University, Jeddah 3270, Saudi Arabia; 4 Department of Food and Nutrition, Faculty of Human Sciences and Design, King Abdulaziz University, Jeddah 3270, Saudi Arabia; 5 Department of Biochemistry, Faculty of Science, King Abdulaziz University, Jeddah 21589, Saudi Arabia

**Keywords:** *ABCA1* C69T gene polymorphism, dysglycemia, dyslipidemia, type 2 diabetes, HDL-cholesterol, triglycerides

## Abstract

Studies suggest that ATP-binding cassette transporter A1 (*ABCA1* C69T) polymorphism is associated with a decreased incidence of type 2 diabetes mellitus (T2DM) and that there is an association between *ABCA1* C69T polymorphism and the risk of dyslipidemia in diabetic individuals. However, other studies contradict these suggestions. Therefore, we aimed to investigate the prevalence of *ABCA1* C69T (rs1800977) gene polymorphism in a representative sample of the Saudi population not previously diagnosed with diabetes and its possible association with dyslipidemia and dysglycemia. A cross-sectional design was used to recruit nondiabetic adults of both genders from the Saudi population in Jeddah by employing a stratified, two-stage cluster sampling method. A total of 650 people (337 men and 313 women) were recruited. Demographic, dietary, and lifestyle variables, as well as medical history and family history of chronic diseases, were collected using a predesigned questionnaire. Fasting blood samples were taken for the determination of fasting plasma glucose (FPG), glycated hemoglobin (HbA1c), and lipids profile, which were followed by a 1-h oral glucose tolerance test (OGTT). Real-time PCR technology was used to determine the *ABCA1* C69T gene SNP (rs1800977). The T allele of *ABCA1* C69T (rs1800977) was very frequent (TT in 44.9% and CT in 43.7%). There was a trend toward significance for a higher dysglycemia percentage in people with CT and TT genotypes (25.7%, and 23.3%, respectively) compared with CC genotypes (16.2%). In addition, FPG and 1-h plasma glucose were significantly higher in people with both TT and CT genotypes compared to CC genotypes. However, T allele was not associated with any dysregulation of lipid parameters.

## 1. Introduction

Type 2 diabetes mellitus (T2DM) is a major global health problem. According to the International Diabetes Federation, prediction reports estimated that T2DM can affect around 600 million people by 2025 [1]. In Saudi Arabia, the prevalence of T2DM in adults had been estimated to be 14.4%, according to the World Health Organization report [2]. In addition, the incidence of prediabetes, which is the condition prior to the onset of T2DM, is significantly increasing [3,4]. Although not all the symptoms required to label a person as diabetic are present in the prediabetes stage, the blood sugar is abnormally high. Various organizations used different criteria to define prediabetes. The World Health Organization (WHO) has defined prediabetes as a state of intermediate hyperglycemia using two specific parameters, impaired fasting glucose (IFG) and impaired glucose tolerance (IGT) [5], while the American Diabetes Association (ADA) added elevated hemoglobin A1c (HbA1c) of 5.7% to 6.4% to the diagnostic criteria of prediabetes [6]. The prediabetes phase can take several years to develop T2DM disease [7,8,9]. The conversion rate of individuals from prediabetes to diabetes changes with population characteristics and the criteria used to define prediabetes [10,11]. In a previous study (Bahijri et al.), age was found to be the strongest predictor of prediabetes, which was followed by obesity and, in particular, abdominal obesity, while family history of diabetes, the presence of hypertension and/or dyslipidemia were not associated with the condition [12].

Several studies have reported the success of lifestyle interventions in preventing diabetes in adults with prediabetes, with a relative risk reduction of 40–70% [13,14,15,16]. However, in most cases, prediabetes remains undiagnosed and hence untreated, allowing further deterioration in metabolic regulation and worsening micro and macro-vascular complications [7,8,9]. Indeed, studies have shown an association between prediabetes and increased risk of complications of diabetes such as early nephropathy, small fiber neuropathy, early retinopathy and risk of macrovascular disease [17,18,19,20,21]. In contrast, an Iranian study reported an association between dyslipidemia and prediabetes [22].

T2DM is a complex multifactorial disease characterized by impaired insulin secretion resulting from pancreatic β-cell dysfunction and/or insulin resistance, with subsequent dysregulation of carbohydrate, lipid and protein metabolisms [22]. T2DM patients develop dyslipidemia, causing high levels of triglycerides (TAG) and low-density lipoprotein (LDL-cholesterol) with low levels of high-density lipoprotein (HDL-cholesterol) [13,14,15,16]. Indeed, numerous epidemiological studies reported that the risk of developing cardiovascular diseases (CVD) for diabetic patients is two to four times higher than for individuals without diabetes [15,16].

The genome-wide association studies in 2007 have revealed many genetic variants, and some groups of single-nucleotide polymorphism (SNP) were related to T2DM, including *SLC30A8, FTO, CDKAL1, CDKN2A, CDKN2B, HHEX, IGF2BP2, GCKR* and *ABCA1* SNPs [17,18,19].

The ATP-binding cassette transporter A1 (ABCA1) is a cell membrane transport protein composed of 2261 amino acids [20]. The main function of ABCA1 is to promote the efflux of cellular cholesterol and phospholipids [21]. In pancreatic β-cells, the interaction of extracellular amphipathic apolipoproteins with ATP-binding cassette transporter 1 (ABCA1) leads to cholesterol efflux and insulin granules fusion and the subsequent liberation of insulin hormone [23]. Therefore, the loss of function of *ABCA1* from β-cells is expected to be related to β-cells dysfunction, causing the level of intracellular cholesterol to be increased and insulin secretion to be compromised [24]. 

The *ABCA1* gene is a highly polymorphic gene located on chromosome 9 [25]. Previous studies suggested that the *ABCA1* C69T polymorphism variant is the upstream variant of *ABCA1* gene (ENST00000374736.8) in the non-coding region shown to be associated with a decreased incident rate of T2DM [26]. In addition, a recent meta-analysis of studies conducted on Asian people (mainly Chinese) reached the same conclusion [20]. However, ethnicity has been reported to play a role in the determination of genetic risk factors [27,28], and indeed, others have reported no effects in Bangladeshi [29], Malaysian [30], or Caucasian subjects [31].

It was also suggested that the genetic polymorphisms of *ABCA1* C69T gene could modulate the *ABCA1* transcription, resulting in reduced HDL levels with an accumulation of cholesterol in the peripheral tissues, which leads to atherosclerosis and dyslipidemia-related disorders [32,33,34]. Recent studies on different populations have observed an association of *ABCA1* C69T polymorphism with the risk of dyslipidemia in diabetic individuals [20]. However, other studies reported no association with dyslipidemia [26,35]. In a recent study investigating the possible association between *ABCA1* C69T gene polymorphism and T2DM in a Saudi population by using a case-control design, the frequency of the T allele of the *ABCA1* C69T gene was significantly higher in healthy subjects compared to T2DM patients [36]. Therefore, it was suggested that the T allele may be a protective factor against T2DM in the Saudi population.

However, there are no published studies carried out on Saudi individuals investigating the association between *ABCA1* C69T gene polymorphism and the risk of dyslipidemia in prediabetics or undiagnosed diabetic individuals. Therefore, the aim of the present study was to investigate the prevalence of *ABCA1* C69T (rs1800977) gene polymorphism in a randomly collected representative sample of the Saudi population not previously diagnosed with diabetes and its possible association with dyslipidemia and dysglycemia.

## 2. Materials and Methods

### 2.1. Study Design

A cross-sectional study was carried out on nondiabetic adults of both genders from the Saudi population recruited from healthcare centers representing all health sectors in Jeddah by employing a stratified, 2-stage cluster sampling method [37]. Those who were diagnosed with diabetes, cardiovascular disease, cancer, kidney or liver diseases, gastrointestinal diseases needing special diet, physical or mental disabilities, as well as pregnant females, were excluded. A total of 724 subjects (370 men and 354 women) were recruited. The study was approved by the Ethics Committee of Human Research at King Abdulaziz University (Reference No. 464–22).

A full explanation of the sampling methodology has been outlined earlier [38] and is summarized here as follows: adults (age ≥18 years) not previously diagnosed with diabetes were recruited from attendees of Primary Health Care (PHC) centers in Jeddah. Following signing an informed consent form, demographic, dietary, and lifestyle variables, as well as medical history and family history of chronic diseases, were collected from recruits using a predesigned questionnaire. Fasting blood samples were taken for the determination of fasting plasma glucose (FPG), glycated hemoglobin (HbA1c), and lipid profile, followed by a 1-h oral glucose tolerance test (OGTT) [39,40] to screen for diabetes and prediabetes. Anthropometric measurements (weight, height, waist circumference (WC)), hip circumference (HP), neck circumference (NC) and blood pressure (BP) were measured using standardized equipment and techniques. Weight and height were used to calculate the body mass index (BMI).

### 2.2. Diagnosing Diabetes, Prediabetes and Dyslipidemia

Prediabetes was defined as HbA1c 5.7–6.4% (39–46 mmol/mol), FPG 6.1–6.9 mmol/L (impaired fasting glucose) or 1 h plasma glucose (1 h PG) 8.6–11.0 mmol/L (impaired glucose tolerance) [38]. Participants with HbA1c ≥ 6.5%, FPG ≥ 7 mmol/L or 1 h OGTT ≥ 11.1 mmol/L were considered to have diabetes [38,41,42,43]. People with either prediabetes or diabetes were considered to have dysglycemia [38]. Dyslipidemia was defined as LDL-C ≥ 3.37 mmol/L, HDL-C < 1.04 mmol/L for men and <1.3 mmol/L for women, total cholesterol (TC) ≥ 5.18 mmol/L, triglycerides ≥ 1.7 mmol/L or treatment with lipid-lowering drugs [44].

### 2.3. General Biochemical Testing

Whole blood, serum and plasma samples were sent regularly to an accredited laboratory by the College of American Pathologists at the National Guard Hospital in Jeddah. Plasma glucose and serum TC, HDL-C, and TG levels were measured by spectrophotometric methods using Architect c8000 auto-analyzer (ABBOTT-USA). LDL-C was calculated using the Friedewald equation [41]. HbA1c was measured with high-pressure liquid chromatography (HPLC) using an automated HbA1c analyzer G8 (TOSOH Corporation-Japan).

### 2.4. Genetic Analysis

#### 2.4.1. Isolation of DNA

Genomic DNA was extracted from whole blood samples using GeneJET Whole Blood Genomic DNA Purification Mini Kit obtained from Thermo Fisher Scientific, USA, according to the manufacturer’s instructions. The DNA concentration and purity were measured by the NanoDrop ND-2000 Series Spectrophotometers at 260/280 nm wavelengths.

#### 2.4.2. TaqMan Genotyping Assays

All the extracted DNA samples were genotyped for rs1800977 polymorphism using TaqMan genotyping assay by a StepOne™ real-time PCR system from Applied Bio-system. The assay included two primers for amplifying the sequence of interest and two TaqMan minor groove binder (MGB) probes for detecting alleles which permit the genotyping of the two possible variant alleles at the SNP site in a DNA target sequence, as the two probe pairs presence in each reaction and the changes in fluorescence of the dyes associated with the probes determine the presence or absence of an SNP. The TaqMan MGB Probes consist of two target-specific oligonucleotides; the first is a reporter dye at the 5′ end of each probe: VIC dye (green fluorophore) is linked to the 5′ end of the wild-type allele labeled, and FAM dye (blue fluorophore) is linked to the 5′ end of the allele mutant allele. The second is a non-fluorescent quencher (NFQ) at the 3′ end of the probe. The production of green fluorescence during amplification implies homozygous wild type; blue fluorescence implies homozygous mutant, whereas a combination of both green and blue fluorescence indicates the heterozygous state. For scatter plot analysis, the automatically generated threshold cycle values from each sample were plotted at coordinates that correspond to the signal of either FAM or VIC. The 10 μL reaction mixture contained 5 μL of TaqMan Genotyping Master Mix (cat no# 4351379), (Assay ID: C__9456257_10) obtained from Thermo Fisher (Thermo Fish-er Scientific, Waltham, MA, USA), which contains AmpliTaq Gold DNA Polymerase UP (Ultra-Pure), dNTP without dUTP, Passive Reference ROX dye and optimized mix components, 0.5 μL TaqMan SNP Genotyping Assay 40X (which includes 300 nM of each specific oligonucleotide primer and MGB probe 200 nM), 1 μL of DNA template corresponding to 10–50 ng/μL and nuclease free water up to volume. The reaction mixture was placed in a Micro Amp optical 96-well reaction plate. The conditions for PCR included 60 °C for 1 min, 95 °C for 10 min followed by 40 cycles of 95 °C for 15 s and 60 °C for 1 min. Fluorescence signal intensity was measured using a StepOne™ real-time PCR system from Applied Biosystem and results were analyzed using its System SDS software.

### 2.5. Statistical Analysis

Data analysis was performed using IBM SPSS statistics version 24.0 for Windows. Baseline characteristics were expressed as mean (SDs) or n (%). Demographic, anthropometric and clinical variables of people with different alleles were compared. A one-way ANOVA or Kruskal–Wallis one-way analysis of variance, as appropriate, was used to compare factors with continuous variables between the 3 groups (CC, CT, and TT), while the Chi-square test was used to compare categorical variables. Differences in dysglycemia and dyslipidemia measurements between every 2 groups were analyzed using the *t*-test or Mann–Whitney test, as appropriate. After adjustment for age, BMI and gender, nominal regression was used to assess association between alleles and dysglycemia and dyslipidemia metabolic variables. A *p* value less than 0.05 (2-sided test) was accepted as statistically significant.

## 3. Results

Complete data were found for 650 subjects, 337 (51.8% of the total) males, and 313 (48.2% of the total) females. 

The genotyping frequencies of *ABCA1* C69T for the three studied groups were in Hardy–Weinberg equilibrium (P >001). The genotypic distribution of *ABCA1* C69T polymorphism was CC in 74 (11.4%), TT in 292 (44.9%) and CT in 284(43.7%) people. The demographic, anthropometric and clinical characteristics of people with different C69T genotype alleles are presented in Table 1. There were no significant differences in these characteristics between C69T genotype alleles.

Examining the lipid profile results, 387 persons (59.5% of the studied population) were found to have dyslipidemia, but only 91 were previously diagnosed and were taking medications if not on a regular basis. In addition, 153 persons (23.5% of the studied population) were found to have dysglycemia (undiagnosed diabetes and prediabetes), but none were taking any medication for the condition. 

No significant differences in the distribution of dysglycemia and dyslipidemia were found between the three genotypes. However, there was a trend toward significance for a higher dysglycemia percentage in people with CT and TT genotypes (25.7% and 23.3%, respectively) when compared with those who have CC genotypes (16.2%) (*p* = 0.083, 0.088, Chi-square test; Table 2). In addition, comparing the means of each component of dysglycemia between different genotypes showed that mean FPG and plasma glucose (1 h) were significantly higher in people with both TT (FPG = 4.48 ± 1.05, and 1 h PG = 6.78 ± 2.13) and CT (FPG = 4.49 ± 0.93, and 1 h PG = 6. 8 ± 2.19) genotypes compared to CC genotype (FPG = 4.28 ± 0.87 and 1 h PG = 6.26 ± 1.88) (FPG: CC vs. TT *p* < 0.01, CC vs. CT *p* < 0.01 and 1 h PG: CC vs. TT *p* < 0.05, CC vs. CT *p* < 0.05; Table 3). However, comparing the means of each component of dyslipidemia between different genotypes showed no statistically significant differences.

TT genotype was significantly associated with increased 1 h PG compared with CC genotype (TT vs. CC: OR-1.931; 95%CI: 1.004–3.715; *p* < 0.05; Table 4). However, after adjustment for age, sex and BMI, this became a trend toward significance (TT vs. CC: OR-1.815; 95%CI: 0.923–3.692; *p* = 0.08; Table 4). There was no association between the genotypes and dyslipidemia or its components (Table 5).

## 4. Discussion

The aim of this present study was to investigate the prevalence of *ABCA1* C69T (rs1800977) gene polymorphism in a randomly collected representative sample of Saudi adults not previously diagnosed with diabetes and to study the possible association of different genotypes with dyslipidemia and dysglycemia. A two-stage, cross-sectional study design was employed to ensure the collection of a representative sample from the local population, since using a case-control design would have biased the results. The inclusion of people not previously diagnosed with diabetes helped to avoid the interference of glucose-lowering medications with measured biochemical markers of dysglycemia. Moreover, those found to have dyslipidemia were mostly not previously diagnosed. Hence, very few people were taking medications on a regular basis.

The frequency of the T allele was high in the studied population, with 88.6% (576 persons) being of TT or CT genotype. No studies on the prevalence of the different alleles among Saudis or other ethnic populations in Saudi Arabia can be found in the literature. All previous studies were conducted using a case-control design, with diabetic or dyslipidemic patients as cases, which does not allow calculation of prevalence. Nevertheless, a relatively high frequency of the T allele was reported in both cases and controls [26,29,30,35,44]. 

Investigating the possible association of different genotypes with dyslipidemia and dysglycemia, we found no significant differences in the distribution of dysglycemia and dyslipidemia between the three genotypes, but there was a trend toward significance for a higher dysglycemia percentage in people with CT and TT genotypes (25.7%, and 23.3%, respectively) when compared with those who have CC genotypes (16.2%) (Table 2). In addition, we found that mean FPG and plasma glucose (1 h) were significantly higher in people with both TT and CT genotypes compared to CC genotype (Table 3). Our results contrast with results from previous studies on Asian population (mainly Chinese) [26,30,42], as well as results from a Saudi study conducted in Riyadh [44] using a case-control design which reported that the TT genotype of the rs1800977 polymorphism was associated with a decreased risk of T2DM compared to the CC genotype. However, studies in other populations reported no effect on T2DM risk [29,41] or increased risk [35]. These differences emphasize the effect of ethnicity on risk assessment. The difference between our results and that of the mentioned Saudi study [44] might be due to different study design, as well as the lack of statistical adjustment for other risk factors for T2DM in the Riyadh study, such as age, gender, family history, and waist circumference, which were all significantly different comparing cases to controls. Adjustment for known risk factors is much needed since T2DM is a multifactorial disease. Indeed, before adjustment, the TT genotype was significantly associated with increased 1 h PG compared with CC genotype (TT vs. CC: OR-1.931; 95%CI: 1.004–3.715; *p* < 0.05; Table 4). However, after adjustment for age, sex, and BMI, this became a trend toward significance (TT vs. CC: OR-1.815; 95%CI: 0.923–3.692; *p* = 0.08; Table 4). 

In an attempt to explain the differences in results reported by different researchers and the difference between our results and that of the Riyadh study, various previous related studies were reviewed. An earlier study on mice reported that the targeted deletion of β-cell *ABCA1* caused an accumulation of cholesterol in islets as well as reduced glucose-stimulated insulin secretion (GSIS) and impaired glucose tolerance [38]. However, it was reported by Vergeer and his group that HDL-c levels in heterozygous carriers of disruptive mutations in *ABCA1* were less than half those in family-based noncarriers of similar BMI, sex, and age, but LDL cholesterol levels did not differ. In addition, FPG was found to be similar between the two groups, but glucose curves after an OGTT were mildly higher in carriers than in noncarriers. Moreover, carriers demonstrated lower first-phase insulin secretion than noncarriers but no difference in insulin sensitivity. Therefore, it was concluded that the level of plasma cholesterol exposed to the islets determines the degree of β-cell dysfunction caused by *ABCA1* deficiency [43]. Admittedly, Vergeer et al. did not investigate the effect of the *ABCA1* C69T polymorphism, but their findings might offer an explanation for the above-mentioned differences in results. Moreover, the report by Vergeer et al. strengthens our findings of increased 1 h PG in TT compared with CC genotype; in particular, the means of the different components of the lipid profile were not significantly different between the three genotypes. 

Investigating the association between the genotypes and dyslipidemia, and after adjustment for age, sex and BMI, we found no association between the genotypes and dyslipidemia or its components (Table 5). Our findings are similar to that reported in another Saudi study [44] as well as other studies in different populations [26,30], which reported that no relationship between *ABCA1* C69T genotypes and lipid profiles was observed [44]. In contrast, an Egyptian study (30) reported that the *ABCA1* TT genotype was associated with hypercholesterolemia and diminished HDL in T2DM patients, which could be due to the associated increased BMI found in this genotype. Similarly, a meta-analysis of studies on different ethnic populations found that the T allele carriers in 69C>T had lower HDL-levels [20]. Another meta-analysis of studies on Caucasians found that compared with that in nondiabetic subjects, the T allele significantly reduced the risk of hypertriglyceridemia in diabetic patients [41]. Differences in the Design studied populations and lifestyle could explain differences in findings, especially since dyslipidemia is strongly associated with lifestyle and diet [45,46].

Our study has some limitations as well as points of strength. The first point of strength is that this study is the first and only study to investigate the prevalence of *ABCA1* C69T (rs1800977) gene polymorphism in Saudi adults not previously diagnosed with diabetes. The second point of strength is that by choosing undiagnosed people with T2DM, in contrast to previous studies which used case-control design, helped to exclude the influence of medications on measures of dysglycemia so that a true association is arrived at between the studied polymorphism and risk of dysglycemia. Another point of strength is the use of regression analysis to adjust for known risk factors for dysglycemia, namely age, sex and BMI. 

The study’s main limitation is the inclusion of many prediabetic people who may or may not eventually develop T2DM while their genotype remains unchanged. However, their inclusion was necessary to study the association between the different genotypes and dysglycemia. A future cohort study following the prediabetic people over a period of time to investigate the progression to diabetes will help to elucidate the true association with T2DM.

Another limitation is the inclusion of some individuals with dyslipidemia who were taking medications which could influence the association of genotypes with dyslipidemia. Reanalysis of data following the exclusion of those taking medications for dyslipidemia and increasing sample size will be carried out in a future study.

## 5. Conclusions

In conclusion, we found that the T allele of *ABCA1* C69T (rs1800977) is very frequent (TT in 44.9% and CT in 43.7%) in our studied Saudi population. This allele was associated with some impairment in insulin secretion in response to glucose load but was not associated with any dysregulation of lipid parameters. A future cohort study will help to clarify the association between this polymorphism and risk of T2DM and or dyslipidemia.

## Figures and Tables

**Table 1:** Demographic, anthropometric and clinical characteristics of the studied population according to their genotype.

	CC (n=74)	TT (n=292)	CT (n=284)
Gender			
Male n (n%)	38 (51.4)	157 (53.8)	142 (50.0)
Female n (n%)	36 (48.6)	135 (46.2)	142 (50.0)
Weight, kg mean (SD)	71.4 (14.3)	74.9 (18.2)	75.3 (18.2)
BMI mean (SD)	26.6 (5.04)	27.6 (6.26)	28 (6.15)
Fat, % mean (SD)	32.6 (11.3)	32.9 (13.2)	34.6 (11.3)
NC, cm mean (SD)	36 (4.05)	36.8 (5.13)	36.6 (6.76)
WC, cm mean (SD)	91.1 (13.8)	92.5 (15.9)	93 (15.8)
HC, cm mean (SD)	104 (12)	106 (14)	106 (14)
WC: HC mean (SD)	0.88 (0.09)	0.85 (0.16)	0.85 (0.16)
SBP mean (SD)	118 (19)	116 (16)	117 (15)
DBP mean (SD)	72 (12)	72(12)	73 (12)

BMI, body mass index; DBP, diastolic blood pressure; HC, hip circumference; NC, neck circumference; SBP, systolic blood pressure; WC, waist.

**Table 2:** Distribution of dysglycemia and dyslipidemia in people with different *ABCA1* C69T genotypes.

Genotypes	*n*	Dysglycemia n (%)	Dyslipidemia n (%)
CC	74	12 (16.2)	39 (52.7)
TT	292	68 (23.3)	171 (58.6)
CT	284	73 (25.7)	177 (62.1)
CC + TT	366	80 (19.8)	210 (55.7)
CC + CT	358	85 (21)	216 (57.4)
TT + CT	576	141 (24.5)	348 (60.4)

Data are reported as number (n) and percentage in each genotype. Associations between dysglycemia and dyslipidemia and *ABCA1* C69T alleles were analyzed by Chi-square. There were no significant associations.

**Table 3:** Comparison of the mean values of clinical variables across the three genotypes of the *ABCA1* C69T gene.

	CC (n = 74)	TT (n = 292)	CT (n = 284)	CC vs. TT *p*-Value ^a^	CC vs. CT *p*-Value ^a^	TT vs. CT *p*-Value ^a^	*p*-Value ^b^
HbA1c %	5.27 (0.55)	5.24 (0.46)	5.28 (0.52)	0.7 ^a2^	0.816 ^a2^	0.317 ^a2^	0.606 ^b2^
FPG	4.28 (0.87)	4.48 (1.05)	4.49 (0.93)	**0.009** ^a2^	**0.009** ^a2^	0.942 ^a2^	**0.022** ^b2^
PG (1 h)	6.26 (1.88)	6.78 (2.13)	6.8 (2.19)	**0.042** ^a2^	**0.049** ^a2^	0.927 ^a2^	0.107 ^b2^
TC (mmol/L)	4.94 (0.95)	4.78 (0.94)	4.85 (0.95)	0.194 ^a2^	0.730 ^a2^	0.224 ^a2^	0.314 ^b2^
HDL-c (mmol/L)	1.35 (0.26)	1.35 (0.28)	1.33 (0.29)	0.88 ^a2^	0.364 ^a2^	0.241 ^a2^	0.43 ^b2^
TG (mmol/L)	1.36 (0.92)	1.21 (0.77)	1.37 (1.02)	0.398 ^a2^	0.844 ^a2^	0.11 ^a2^	0.262 ^b2^
LDL (mmol/L)	3.31 (0.86)	3.2 (0.84)	3.25 (0.86)	0.334 ^a2^	0.818 ^a2^	0.265 ^a2^	0.437 ^b2^
LDL:HDL	2.56 (0.9)	2.46 (0.76)	2.56 (0.87)	0.732 ^a1^	0.785 ^a1^	0.300 ^a1^	0.59 ^b1^

Data are presented as mean (SD). FPG, fasting plasma glucose; HbA1c, glycated hemoglobin; HDL-c, high-density lipoprotein cholesterol; LDL-c, low-density lipoprotein cholesterol; PG, plasma glucose; TC, total cholesterol; TG, triglycerides. ^a^ Differences in measurements were analyzed using the *t*-test (^a1^) or Mann–Whitney test (^a2^). ^b^ Differences in measurements were analyzed using the one-way ANOVA (^b1^) or Kruskal–Wallis (^b2^) test. Bold indicates significant differences in mean values.

**Table 4:** Logistic regression analysis of dysglycemic abnormalities and the three genotypes of the *ABCA1* C69T gene.

	Normal	Abnormal	Unadjusted OR (95% CI)	Adjusted for Age, BMI and Gender OR (95% CI)
**Dysglycemia**				
CC	62 (83.8)	12 (16.2)	reference	reference
TT	224 (76.7)	68 (23.3)	1.788 (0.912, 3.504)	1.39 (0.683, 2.828)
CT	221 (74.3)	73 (25.7)	1.568 (0.799, 3.081)	1.638 (0.807, 3.325)
**FPG**				
CC	70 (94.6)	4 (5.4)	reference	reference
TT	266 (93)	20 (7)	1.316 (0.436, 3.974)	1.047 (0.338, 3.24)
CT	259 (92.2)	22 (7.8)	1.486 (0.496, 4.455)	1.202 (0.392, 3.692)
**PG (1 h)**				
CC	57 (81.4)	13 (18.6)	reference	reference
TT	193 (69.4)	85 (30.6)	**1.931 (1.004, 3.715)**	1.815 (0.923, 3.57)
CT	198 (73.9)	70 (26.1)	1.55 (0.8, 3.003)	1.463 (0.738, 2.899)
**HbA1C (%)**				
CC	65 (90.3)	7 (9.7)	reference	reference
TT	243 (88)	33 (12)	1.261 (0.533, 2.981)	1.047 (0.429, 2.558)
CT	232 (84.4)	43 (15.6)	1.721 (0.739, 4.006)	1.458 (0.429, 2.558)

Data are presented as n (%). FPG, fasting plasma glucose; HbA1c, glycated hemoglobin; PG, plasma glucose. Significant differences between genotypes are shown in bold font.

**Table 5:** Logistic regression analysis of dyslipidemic abnormalities and the three genotypes of the *ABCA1* C69T gene.

	Normal	Abnormal	UnadjustedOR (95% CI)	Adjusted for Age, BMI and Gender OR (95% CI)
**Dyslipidemia**				
CC	35 (47.3)	39 (52.7)	reference	reference
TT	121 (41.4)	171 (58.6)	1.268 (0.76, 2.117)	1.176 (0.691, 2.001)
CT	108 (37.9)	177 (62.1)	1.471 (0.879, 2.462)	1.349 (0.79, 2.303)
**TC**				
CC	50 (67.6)	24 (32.4)	reference	reference
TT	191 (67)	94 (33)	1.025 (0.594, 1.77)	0.943 (0.537, 1.657)
CT	187 (66.3)	95 (33.7)	1.058 (0.613, 1.77)	0.98 (0.558, 1.721)
**LDL-c**				
CC	44 (62.2)	30 (37.8)	reference	reference
TT	180 (61.1)	105 (38.9)	1.048 (0.619, 1.775)	0.792 (0.465, 1.351)
CT	163 (58.9)	118 (41.4)	1.145 (0.676, 1.939)	0.997 (0.586, 1.697)
**TG**				
CC	60 (81.1)	14 (18.9)	reference	reference
TT	235 (82.5)	50 (17.5)	0.912 (0.473, 1.759)	0.749 (0.377, 1.491)
CT	213 (75.9)	68 (24.1)	1.362 (0.716, 2.589)	1.194 (0.609, 2.339)
**HDL-c**				
CC	63 (81.1)	11 (18.9)	reference	reference
TT	226 (76.8)	59 (23.2)	1.292 (0.679, 2.458)	1.233 (0.643, 2.365)
CT	211 (73)	70 (27)	1.581 (0.835, 2.994)	1.495 (0.783, 2.854)

Data are presented as n (%). HDL-c, high-density lipoprotein cholesterol; LDL-c, low-density lipoprotein cholesterol; TC, total cholesterol; TG, triglycerides.

## Data Availability

The datasets analyzed for this study can be found at the king Abdulaziz university repository at https://sdrg.kau.edu.sa/Show_files.aspx?Site_ID = 30504&Lng = EN (accessed on 18 October 2022).
